# Exploring the association between epilepsy and depression: A systematic review and meta-analysis

**DOI:** 10.1371/journal.pone.0278907

**Published:** 2022-12-15

**Authors:** Shao-kun Qin, Zi-xian Yang, Zhen-wei Guan, Jin-hu Zhang, Xin Ping, Ye Lu, Lin Pei

**Affiliations:** 1 Hebei University of Chinese Medicine, Shijiazhuang, Hebei, China; 2 Hebei Key Laboratory of Turbidity, Shijiazhuang, Hebei, China; 3 School of Chinese Medicine, North China University of Science and Technology, Tangshan, Hebei, China; 4 Hebei Academy of Chinese Medicine Sciences, Shijiazhuang, Hebei, China; UCSI University, MALAYSIA

## Abstract

**Objective:**

This study offers meta-analytic data on the potential association between epilepsy and depression especially for the prevalence of depression in epilepsy or vice versa.

**Methods:**

The relevant studies were searched and identified from nine electronic databases. Studies that mentioned the prevalence and/or incidence of epilepsy and depression were included. Hand searches were also included. The search language was English and the search time was through May 2022. Where feasible, random-effects models were used to generate pooled estimates.

**Results:**

After screening electronic databases and other resources, 48 studies from 6,234 citations were included in this meta-analysis. The period prevalence of epilepsy ranged from 1% to 6% in patients with depression. In population-based settings, the pooled period prevalence of depression in patients with epilepsy was 27% (95% CI, 23–31) and 34% in clinical settings (95% CI, 30–39). Twenty studies reported that seizure frequency, low income, unemployment of the patients, perception of stigma, anxiety, being female, unmarried status, disease course, worse quality of life, higher disability scores, and focal-impaired awareness seizures were risk factors for depression.

**Conclusion:**

Our study found that epilepsy was associated with an increased risk of depression. Depression was associated with the severity of epilepsy.

## Introduction

Epilepsy is a common neurological disorder in which abnormal electrical discharges in the brain can lead to recurrent seizures [1]. Epileptic seizures are generally rare with an annual incidence of approximately 0.3‰ for newly diagnosed epilepsy and 0.55‰ for unprovoked seizures [2]. In epilepsy, depression is the most common psychiatric comorbidity. Depression affects around one‐third of these cases and impacts quality of life [3]. Depression is the most common psychiatric disorder, and it occurs in 14.1% of females and 14.8% of males worldwide [4]. Depression is more frequent in patients with epilepsy compared to the general population [5]. Epilepsy and depression both can influent individual’s interpersonal communication, social activities and can increase the risk of sudden attacks [4,6]. Some studies indicate that epilepsy and depression are bidirectional [7]. The reported prevalence of depression in patients with epilepsy (PWE) varies between 10.7 to 44%, and it can reach 54% in refractory epilepsy [8]. However, the association between depression and epilepsy have not yet been comprehensively described.

The epidemiology and risk factors of depression in patients with epilepsy are unclear and vice versa. Understanding the epidemiology of depression and epilepsy is important in reducing disability and protecting patients’ health and safe. Our study offers a comprehensive and systematic review of the prevalence, incidence, and reported risk factors for depression with epilepsy and epilepsy with depression. We further studied direct associations between depression and epilepsy.

## Methods

### Protocol and registration

We registered this systematic review on the Prospective Register of Systematic Reviews (PROSPERO) on April, 2022 (#CRD42022327256). This systematic review and meta-analyses were reported with a predetermined protocol and the Preferred Reporting Items for Systematic Reviews and Meta-Analyses (PRISMA) statement.

### Information sources

**Fig 1:**
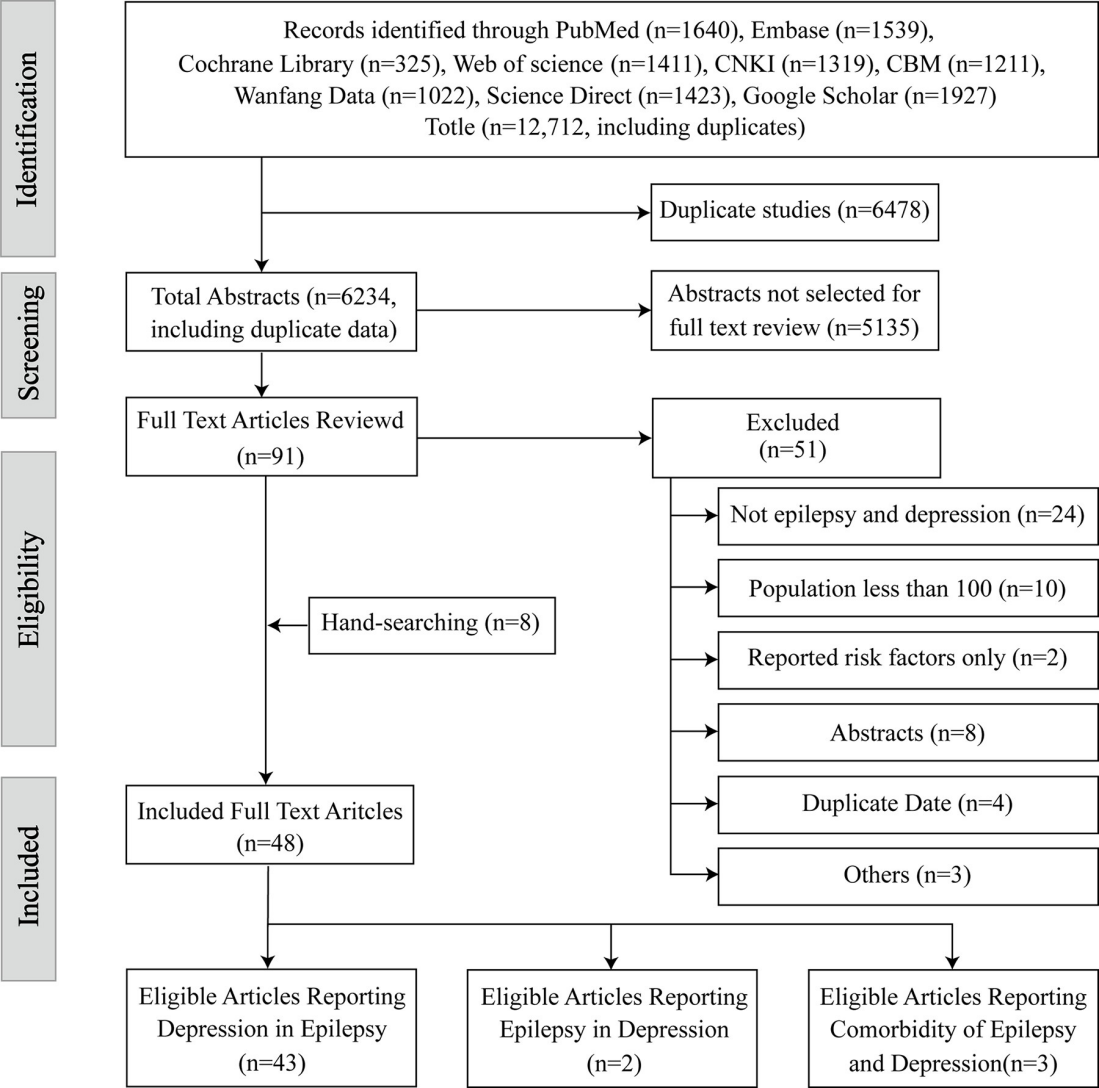
PRISMA flow diagram.

Nine databases were searched from inception to May 15, 2022 (Fig 1). EndNote X9 was used to export and manage references. Search terms included awakening epilepsy, epilepsia, epileptic, epilepticus, seizure disorder, epilepsy, cryptogenic epilepsies, cryptogenic epilepsies, aura, depression, depressive symptoms, symptom depressive, emotional depression, etc. In addition, reference lists and bibliographies from cited documents were manually searched for additional articles. Hand searches were also included. The search language was English. A complete description of our search strategy is available as a S1 File.

### Study selection

Two reviewers independently studied the titles, abstracts, and full text reviews to find potentially eligible reviews. The eligibility criteria included the following: (a) a clearly recognized diagnostic criteria for epilepsy and depression; (b) a sample size over 100; (c) reported prevalence or incidence of epilepsy in depression, vice versa, or both; the data had to be able to be extracted. The exclusion criteria included the following: (a) reporting only risk factors; (b) no focus on epilepsy and depression; or (c) duplicated studies. The most comprehensive version was selected from duplicate data. Disagreements between reviewers were solved by discussion. If agreement could not be reached, then a third senior study author resolved the issue.

### Data extraction and study quality

**Fig 2:**
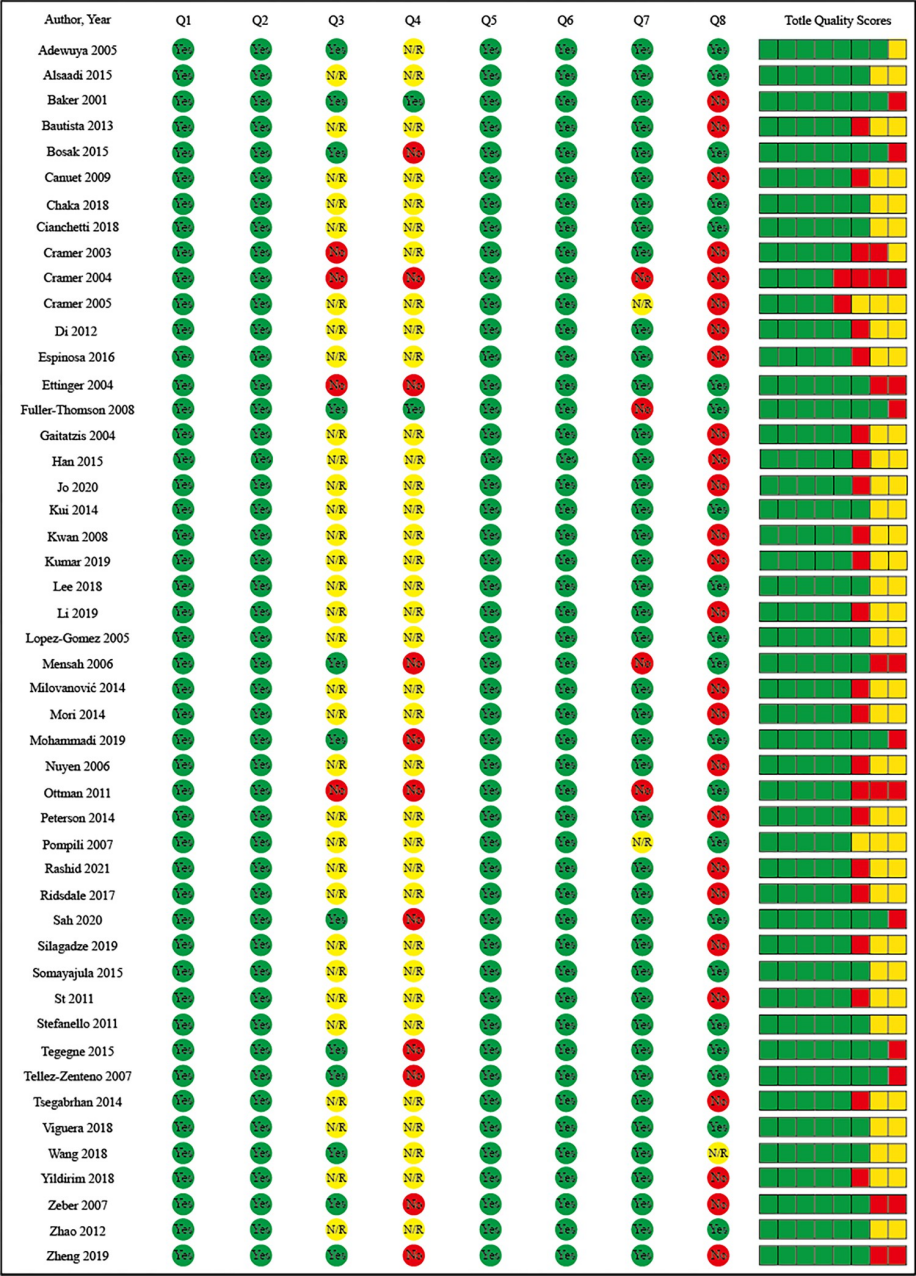
The quality scores of included studies. Q1: Target Population described? Q2: Cases from entire population or probability sampling? Q3: Response rate >70%? Q4: Non-responders clearly described? Q5: Is the sample representative of the population? Q6: Were data collection methods standardized? Q7: Were validated criteria used to assess the presence/absence of disease? Q8: Are the estimates of prevalence and incidence given with confidence intervals?

Two authors extracted data independently in duplicate using a standard data abstraction form. Data were extracted by two authors, and the details were as follows: authors and study country, sample size, case size, mean age, age range, female, epileptic diagnostic criteria, depressive diagnostic criteria, data collection period, and prevalence. Research quality indicators related to sample representativeness, conditional evaluation, and statistical methods were extracted and provide the basis for conditional heterogeneity evaluation. Assessments of study quality were performed according to Subota et al [9]; see Fig 2.

### Data synthesis and analysis

Depression in epilepsy, epilepsy in depression, or both were analyzed separately for each condition. The Cochrane Q statistic was calculated to assess the significance between study heterogeneity, and I^2^ was used to quantify the magnitude of between-study heterogeneity. When I^2^<50%, the pooled estimate and 95% confidence intervals (CIs) were calculated using a fixed-effect model. A random-effects model was used when I^2^>50%. Subgroup analysis was performed by sample resource and diagnostic criteria of depression. Our main outcomes were prevalence, confidence intervals, and percentage prevalence. All analyses were completed using Review Manager 5.4.

## Results

There were 12,712 studies preliminarily assessed for eligibility; 6,478 duplicate studies were excluded from Endnote X9. Here, 91 studies were screened at the full-text levels, and 48 studies were included. The reason for elimination was that they did not report depression or epilepsy (n = 24), were composed of a study sample of less than 100 (n = 10), reported duplicate data (n = 4), only reported risk factors of depression or epilepsy (n = 2), only contained an abstract (n = 8), or other reasons (n = 3). Manually checking the reference lists led to eight articles included in the systematic review for a total of 48 (Fig 1). The prevalence of epilepsy in depression was included in two articles (Table 1), the prevalence of depression in epilepsy was included in 43 articles (Table 2), and three articles recorded the comorbid relationship between epilepsy and depression (Table 3).

**Table 1:** Studies reporting on the prevalence and incidence of epilepsy in depression (n = 2).

Author, year (country, region)	Sample (n)	Case (n)	Age (year)	Female (n)	Diagnostic criteria epilepsy	Diagnostic criteria depression	Years of data collection	Prevalence
Mohammadi 2019 IRAN [10]	521	29	6–18	313	NR	NR	2016–2017	5.6%
Nuyen 2006 The Netherlands [11]	6641	50	NR	4452	diagnosis by general practitioner	NR	NR	0.75%

**Table 2:** Studies reporting on the prevalence and incidence of depression in epilepsy (n = 43).

Author, year (country, region)	Sample (n)	Case (n)	Age (year)	Female (n)	Diagnostic criteria epilepsy	Diagnostic criteria depression	Years of data collection	Prevalence
Adewuya 2005 Nigeria [12]	102	29	12–18	37	NR	DSM-Ⅳ	NR	28.43%
Alsaadi 2015 United Arab Emirates [13]	186	50	18–65	105	NR	PHQ-9	2014.9–2015.1	26.88%
Baker 2001 UK [14]	669	163	NR	345	Physician diagnosis	HAD	NR	24.36%
Bautista 2013 USA [15]	200	71	≥18	156	NR	NIDDI-E	2012.6–2012.8	35.50%
Bosak 2015 Poland [16]	289	84	NR	49	NR	BDI	NR	29.06%
Canuet 2009 Japan [17]	114	51	18–80	49	Imaging	BDI-II	2006.6–2008.5	44.73%
Chaka 2018 Ethiopia [18]	422	185	≥18	173	NR	PHQ-9	2015.4–2015.5	43.83%
Cianchetti 2018 Italy [19]	326	30	8–18	171	NR	SAFA-D	NR	9.20%
Cramer 2003 USA [20]	683	240	NR	NR	NR	CES-D	2001.1	35.13%
Cramer 2004 USA [21]	649	206	≥18	NR	Seizure Severity Scale and QOLIE-89	CES-D	2001.1	31.74%
Cramer 2005 USA [22]	201	74	19–75	113	QOLIE10	HADS	NR	36.81%
Di 2012 Spanish [23]	121	25	≥18	80	Imaging	MINI	NR	20.66%
Espinosa 2016 Colombia [24]	220	86	18–79	106	ILAE 2014	NDDI-E	2014.9–2015.3	39.09%
Ettinger 2004 USA [25]	775	283	>18	365	Self-reported prior diagnosis of epilepsy	CES-D	2001.1–2002.4	36.51%
Fuller-Thomson 2009 Canada [26]	781	110	≥12	401	A health professional	CIDI-SF	2000–2001	14.08%
Gaitatzis 2004 UK [27]	5834	1063	≥16	NR	ICD-9	NR	1995.1.1–1998.12.31	18.22%
Han 2015 Korea [28]	391	267	18–79	187	NR	BDI	NR	68.28%
Jo 2020 Korea [29]	126	38	>18	62	ILAE 2014	PHQ-9	2018.10–2018.12	30.15%
Kui 2014 China [30]	215	65	≥18	84	ILAE	DSM-Ⅳ	NR	30.23%
Kwan 2009 China [31]	247	94	18–76	133	NR	HADS	2007.3–2007.6	38.05%
Lee 2018 Korea [32]	141	60	>18	68	ILAE 2014	HADS	NR	42.55%
Li 2019 China [33]	461	138	≥18	248	ILAE	CNDDI-E	2017.1–2017.11	29.93%
Lopez-Gomez 2005 Mexico [34]	241	103	NR	116	ILAE	BDI	2002.3–2003.3	42.73%
Mensah 2006 UK [35]	499	139	18–78	252	NR	HADS	NR	27.85%
Milovanović 2014 Serbia [36]	203	67	18–65	118	ILAE 2001	BDI-II	NR	33.00%
Mori 2014 Japan [37]	463	85	≥16	247	NR	IDS-SR	2009.10.2–2011.4.1	18.35%
Ottman 2011 USA [38]	3488	1134	≥18	2125	NR	NR	2008.1–2008.4	32.51%
Peterson 2014 Australia [39]	279	80	≥18	165	NR	HADS	NNR	28.67%
Pompili 2007 Italy [40]	103	43	19–78	72	NR	BDI	NR	41.74%
Rashid 2021 India [41]	449	180	18–75	219	ILAE	MINI	2018.1–2020.3	40.08%
Ridsdale 2017 UK [42]	403	113	16–85	219	QOLIE-31	HADS	NR	28.03%
Silagadze 2019 Georgia [43]	130	31	18–56	68	NDDI-E ILAE	ICD-10	NR	23.84%
Somayajula 2015 India [44]	165	27	>16	62	ILAE	ICD-10	2011.5–20014.4	16.36%
Stefanello 2011 Brazil [45]	153	32	≥13	NR	ILAE	HAD	2006.5–2007.12	20.91%
Tegegne 2015 Ethiopia [46]	415	136	≥18	186	NR	HADS	2013.4–2013.5	32.77%
Tellez-Zenteno 2007 Canada [47]	253	44	≥15	NR	An interviewer asking directly	DSM-IV	2002.5–2002.12	17.39%
Tsegabrhan 2014 Ethiopia [48]	300	148	>18	117	NR	BDI-II	2012.8–2012.10	49.33%
Viguera 2018 USA [49]	1763	584	≥18	952	ICD-9-CM	PHQ-9	2007.10.1–20128.13	33.12%
Wang 2018 China [50]	458	241	≥18	100	NR	C-NDDI-E	NR	52.62%
Yildirim 2018 Turkey [51]	302	139	15–73	170	NR	BDI	NR	46.02%
Zeber 2007 USA [52]	13699	2961	≥18	503	ICD-9	ICD-9	1996.10.1–1999.9.30	21.61%
Zhao 2012 China [53]	140	36	15–71	70	ILAE 1989	HAMD	2007.8–2008.2	25.71%
Zheng 2019 China [54]	184	36	≥18	84	ILAE 2001	BDI	2014.6–2016.1	19.56%

Abbreviation: ILAE = International League Against Epilepsy; QOLIE = the Quality of Life in Epilepsy Inventory; ICD = International Classification of Diseases; DSM-IV = Diagnostic and Statistical Manual of Mental Disorders; BDI = Beck Depression Inventory; HADS = Hospital Anxiety and Depression Scale; PHQ = the patient health questionnaire; CES-D = the Center for Epidemiologic Studies Depression scale; CIDI-SF = Composite International Diagnostic Interview short‐form; NDDI-E = Neurological Disorders Depression Inventory for Epilepsy; CNDDI-E = Chinese version of the Neurological Disorders Depression Inventory for Epilepsy; IDS-SR = the Inventory of Depressive Symptomatology Self Report.

**Table 3:** Studies reporting on the prevalence and incidence of depression and epilepsy (n = 3).

Author, year (country, region)	Sample (n)	Case (n)	Age (year)	Female (n)	Diagnostic criteria epilepsy	Diagnostic criteria depression	Years of data collection	Prevalence
Kumar 2019 USA [55]	120	69	≥18	81	A self‐reported diagnosis of epilepsy	DSM-5	NR	57.70%
Sah 2020 Nepal [56]	142	44	18–68	55	clinically confirmed epilepsy	HAMD	2018.4–2018.9	30.98%
St 2011 Canada [57]	7253	2044	0.03–96	3481	ICD-9-CM ICD-10-CA	NR	1996.4.1–2004.3.31	28.18%

ICD-9-CM, the International Classification of Diseases, Version 9, Clinical Modification; ICD-10-CA, the International Statistical Classification of Diseases and Related Health Problems, Tenth Revision, Canada; DSM-5, Diagnostic and Statistical Manual of Mental Disorders, Fifth Edition; HAMD, Hamilton Depression Scale.

### Epilepsy in depression

Two studies reported the prevalence of epilepsy in patients with depression—one from the Netherlands and one from Iran. Both studies used data from an administrative database. One study reported both the incidence rate of depression in epilepsy and the incidence rate of epilepsy in depression [11]; the other reported the rates of depression in children and adolescents in Iran [10]. There were relatively few studies, and the aggregated overall prevalence was not calculated.

### Depression in epilepsy

Forty-three papers reported a prevalence estimate for depression with epilepsy. The 43 included studies from the United States (n = 8), China (n = 7), the United Kingdom (n = 4), Ethiopia (n = 3), Korea (n = 3), Canada (n = 2), India (n = 2), Japan (n = 2), Australia (n = 1), Brazil (n = 1), Colombia (n = 1), Georgia (n = 1), Italy (n = 1), Mexico (n = 1), Nigeria (n = 1), Poland (n = 1), Serbia (n = 1), Spain (n = 1), Turkey (n = 1), and the United Arab Emirates (n = 1). Among the 43 reports on the incidence rate of depression in epilepsy, 21 describe the demographic and clinical characteristics of epileptic patients in detail (Table 4).

**Table 4:** Demographic and clinical characteristics of epileptic patients (n = 21).

Author, year (country, region)	Sample (n)	Female (%)	Education (%)		Unemployment (%)	Unmarried (%)
			Less than high school	High school and above		
Bautista 2013 USA [15]	200	63.60	23.60	76.40	NR	40.30
Bosak 2015 [16]	289	58.50	31.10	68.90	61.50	56.40
Chaka 2018 [18]	422	40.90	80.80	19.20	9.70	50.20
Cramer 2005 USA [22]	201	56.20	18.20	81.80	17.30	NR
Espinosa 2016 Colombia [24]	220	48.10	NR		NR	85.50
Fuller-Thomson 2009 Canada [26]	781	51.40	40.90	59.10	NR	48.90
Han 2015 Korea [28]	391	47.80	19.10	80.90	37.10	54.10
Jo 2020 Korea [29]	126	48.40	NR		25.40	NR
Kui 2014 China [30]	215	39.00	65.10	34.90	34.80	39.10
Lee 2018 Korea [32]	141	48.20	22.00	78.00	14.20	51.80
Li 2019 China [33]	461	53.80	42.80	57.20	58.00	65.90
Milovanovć 2014 Serbia [36]	203	58.10	23.10	76.90	25.10	58.10
Peterson 2014 Australia [39]	279	59.10	47.30	52.70	51.60	41.60
Ridsdale 2017 UK [42]	403	54.20	47.50	52.50	49.20	51.00
Silagadze 2019 Georgia [43]	130	52.30	66.20	33.80	NR	NR
Somayajula 2015 India [44]	165	37.60	59.40	40.60	6.70	62.40
Tegegne 2015 [46]	415	44.80	82.40	17.60	NR	61.40
Tsegabrhan 2014 Ethiopia [48]	300	39.00	55.70	44.30	21.70	53.30
Wang 2018 China [50]	458	40.60	87.10	12.90	NR	25.80
Yildirim 2018 Turkey [51]	302	56.00	58.00	42.00	26.00	55.00
Zheng 2019 China [54]	184	45.65	61.40	38.60	26.60	47.20

**Fig 3:**
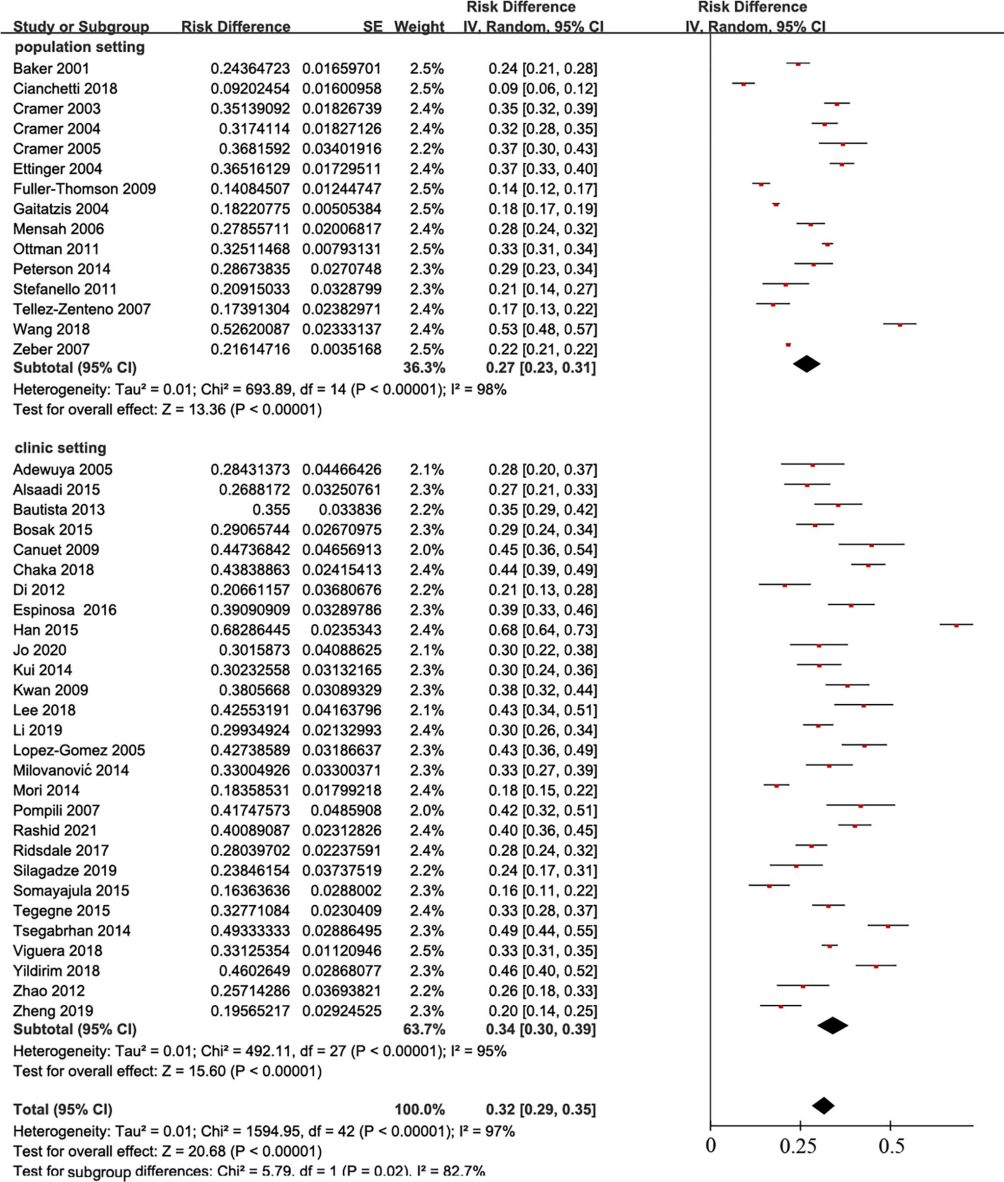
Overall prevalence of depression among persons with epilepsy in population setting and clinic setting.

Among the 43 reports on the incidence rate of depression in epilepsy, 15 were based on a population survey [14,19–22,25–27,35,38,39,45,47,50,52], and 28 were clinical studies [12,13,15–18,23,24,28–34,36,37,40–44,46,48,49,51,53,54]. In a population-based environment, the combined prevalence of epilepsy in depression patients was 27% (95% CI, 23–31), while the prevalence was 34% in the clinic (95% CI, 30–39) (Fig 3).

**Fig 4:**
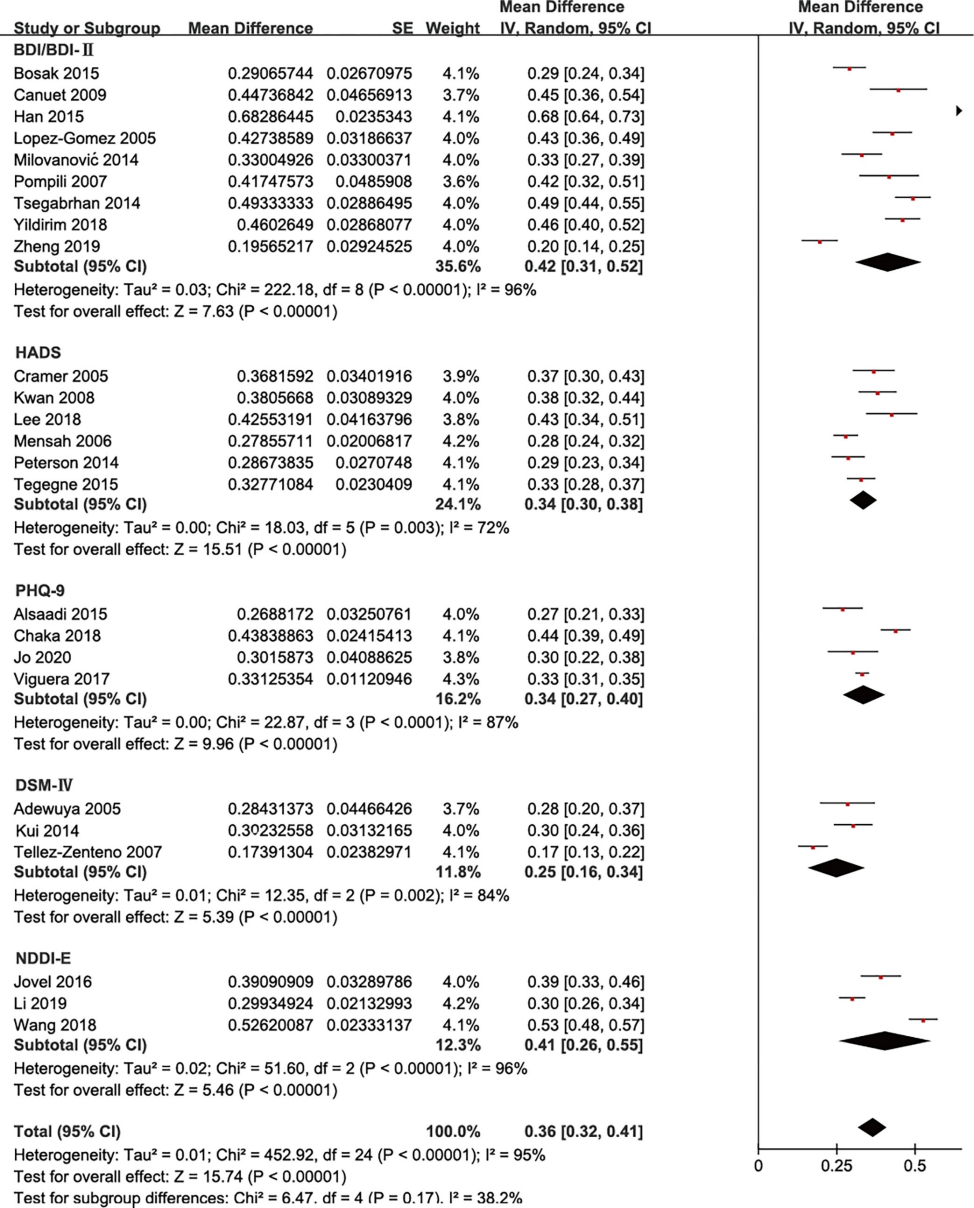
Overall prevalence of depression among persons with epilepsy by different depression diagnostic tool.

Depression is diagnosed with different scales: Beck Depression Inventory (BDI and BDI-Ⅱ), Diagnostic and Statistical Manual of Mental Disorders (Fourth Edition (DSM-Ⅳ)), Hospital Anxiety and Depression Scale (HADS), Neurological Disorders Depression Inventory for Epilepsy (NDDI-E), Patient Health Questionnaire nine-item (PHQ-9), etc. The estimates of depression included here had significant subgroup differences (*P*<0.0001, I^2^ = 94.5%). However, there is no significant subgroup difference when eliminating scales used only once or twice (Fig 4).

Twenty studies explored the risk factors of depression in patients with epilepsy (Table 4). Seizure frequency, low income, unemployment, perception of stigma, anxiety, being female, unmarried status, disease course, worse quality of life, higher disability scores, and focal-impaired awareness seizures were risk factors for depression. Eight studies identified seizure frequency as risk factors for depression in PWE [18,19,24,37,39,46,50,53]. Eight of the articles reported that lower income and unemployment are associated with depression in PWE [10,19,31,34,35,39,45,46]. Six studies found that the perception of stigma was associated with depression in PWE [13,16,19,37,46,53].

### Comorbid epilepsy and depression

Three studies reported the comorbidity of epilepsy and depression [55–57]. Geographically, the three studies were from the United States, Nepal, and Canada. One study acquired data through registries, and two utilized a hospital clinic review. The aggregated overall prevalence was not estimated because of low sample size. One study examined risk factors and found that drug use remained an important predictor of depression among patients with epilepsy (*P* = 0.002); the odds of having depression in patients receiving polytherapy were 3.82-fold higher than in those receiving monotherapy (95% CI, 1.61–9.05, *P* = 0.002) [56].

## Discussion

The median incidence of epilepsy was 50.4 per 100,000 every year ([IQR] 33.6–75.6) [58]; it is estimated that 3.8% of the population suffer from depression. There were more studies on the incidence of depression with epilepsy than epilepsy with depression. This may because depression predicts a worse response to treatment during epilepsy [59] and because people with depression face greater suicide risk [60]; thus, many studies focus on depression with epilepsy. Three studies reported comorbidity, but they do not have specific samples or cases. Few studies reported on occurrence of epilepsy with depression and comorbidity, and a pooled overall prevalence evaluation could not be calculated.

This association may be causal or there may be common pathogenic mechanisms underlying depression and epilepsy. Depression is the most common psychiatric comorbidity in patients with epilepsy [61], and it may explain the worse response to epilepsy treatment [62].

Epilepsy is associated with increasing incidence of depression. Our meta-analysis found that the pooled overall period prevalence of depression in epilepsy based on population (27%, 95% CI, 0.23–0.31) was lower than that based on clinical evaluation (34%, 95% CI, 0.30–0.39). The difference between them was statistically significant (*P* = 0.01). The diagnosis of depression is based on many different scales [63]. Epilepsy is diagnosed through the patient’s clinical symptoms as well as imaging and electroencephalogram changes [64]; however, some population-based diagnoses of epilepsy use questionnaires. We believe that the diagnosis of epilepsy based on clinical features is more accurate than population settings. This condition can perhaps explain the big heterogeneity in the findings because epilepsy was associated with depression [65].

We did subgroup analysis for scales of depression to estimate whether different depression scales affect the above results. The subgroup analysis showed different incidence rates using different scales. These range from 30% to 34% and were statistically significant (*P*<0.0001). However, we do not believe that this difference represents a difference in the detection rates of these scales. First, the I^2^ of the results is 97%. Second, some scales were only used once in our cohort of papers. However, some studies have shown that the clinical use of NDDI-E, HADS, and other scales is not the main driver for these inconsistent results [41]. Thus, we eliminated studies that included these factors and obtained more reasonable results, i.e., no statistical difference between subgroups (Fig 4).

A meta-analysis reported that epilepsy was associated with an increased risk of depression [66]. Risk factors for depression in epilepsy were summarized based on the literature to further investigate the factors influencing the association between epilepsy and depression. There were 20 studies reporting risk factors about depression in epilepsy (Table 5). Seizure frequency, low income, unemployment, and perception of stigma were associated with depression in PWE. A study found that seizure frequency (*P* = 0.36) was not associated with depression [13]. This article did not limit the disease course during the inclusion criteria for patients with epilepsy, which is the main factor influencing depression in patients with epilepsy.

**Table 5:** Risk factors of depression with epilepsy (n = 20).

Author, year	Sample (n)	Age range studied	Risk factors	Examined factors	Statistical method	Depression scales
Adewuya 2005 [12]	102	12–18	Frequency of seizures, number of antiepileptic drugs, perception of stigma	Age, gender, level of education/class, age of onset of illness, duration of epilepsy, seizure type, types of AEDs, number of AEDs	Regression analysis	DSM-Ⅳ
Alsaadi 2015 [13]	186	18–65	Age, gender	Marital status, nationality, seizure frequency age, gender, epilepsy classification, number of seizures in the 6 months prior to the clinic visit	Multi regression mode	PHQ-9
Bosak 2015 [16]	289	NR	Age, frequent seizures, use medications	Age, gender, marital status, education level, occupational activity, use of antidepressant	Logistic regression modeling	BDI
Chaka 2018 [18]	422	≥18	Female, single, perceived stigma, medication adherence, current substance use	Age, gender, ethnicity, marital status, religion, residence, education, occupation, with whom living now	Logistic regression analysis	PHQ-9
Cianchetti 2018 [19]	326	8–18	Severity and duration of the epilepsy	Sex, education, epilepsy severity, disease duration, antiepileptic treatment	Chi-square or Fisher’s exact test	SAFA-D
Espinosa 2016 [24]	220	18–79	Unemployed	Age, sex, education, marital status, and occupational activity, risk factors for epilepsy, age of diagnosis, type of seizures, frequency of seizures, treatment with antiepileptic drugs, and therapeutic response	A multiple linear regression model	NDDI-E
Kui 2014 [30]	215	>18	Employment status, presence of chronic medical illnesses, drug responsiveness	Education, marriage status, employment status, gender, age, age at seizure onset, duration of epilepsy, seizure type, aetiology of epilepsy, epileptic family history, previous status epilepticus, EEG findings, neuroimaging findings outcome of epilepsy, chronic medical illnesses	A binary logistic regression	DSM-Ⅳ
Lee 2018 [32]	141	>18	Higher neuroticism, lower self-esteem, marital status, and lower extroversion	Gender, age at the first seizure onset, marriage, job, economic class, presence vs. absence of perceived stigma	Stepwise linear regression model	HADS
Lopez-Gomez 2005 [34]	241	NR	Seizure frequency	Age, gender, marital status, educational degree, or type of economic activity	A logistic regression model	BDI MADRS
Mensah 2006 [35]	499	18–78	Unemployment	Gender, marital status, or monotherapy or polytherapy antiepileptic medication	A stepwise multiple regression analysis	HADS
Milovanović 2014 [36]	203	18–65	Educational level	Age, educational level, occupational status, marital status, epilepsy history, seizure types, seizure frequency, comorbidity, drug treatment	Hierarchical multiple regression analysis	BDI-II
Peterson 2014 [39]	279	≥18	Employment status, high levels of social stigma, ineffective control of seizures	Gender, employment, marital status, education	Pearson correlations and block recursive regression	HADS
Somayajula 2015 [44]	165	>16	Married	Gender, married, unemployment, graduate age	Logistic regression	ICD-10
Stefanello 2011 [45]	153	≥13	Unemployment, fewer years of schooling, age above 41	Age, gender, marital status, occupation schooling, economic group	Logistic regression analysis	HAD
Tegegne 2015 [46]	415	≥18	Using poly-therapy of anticonvulsants, perceived stigma, inability to read or write	Age, gender, marital status, residence, religion, ethnicity, educational status, occupation, monthly income, frequency of seizure	Logistic regression analysis	HADS
Tsegabrhan 2014 [48]	300	>18	Epilepsy‑related perceived stigma, high seizure frequency, low educational status	Age, duration of illness, marital status, educational status, occupation, place of residence, seizure frequency, type of AEDs, epilepsy‑related perception of stigma	Bivariate logistic regression	BDI‑II
Viguera 2018 [49]	1763	≥18	Age, black race, lower income, lower health-related quality-of-life, higher LSSS score (worse severity)	Age, gender, race, marital status, household median income, patient-reported health-related quality of life, disease-specific performance scale	Univariate logistic regression models	PHQ-9
Wang 2018 [50]	458	≥18	Income, frequent seizures	Gender, marital status, age, income, education, age at seizure onset, polytherapy	NR	C-NDDI-E
Yildirim 2018 [51]	302	15–73	Female, lower education and income levels, never employed, higher seizure frequency	Gender, marital status, educational level, occupation, income level, seizure frequency, seizure type, medication, family history of epilepsy	A multivariate linear regression	BDI
Zhao 2012 [53]	140	15–71	Complex partial seizures, number of seizure types	Gender, seizure type, seizure frequency, number of anti-epilepsy drugs	NR	HAMD

Although there are fewer studies reporting the incidence of epilepsy with depression in our meta-analysis, some studies suggest that depression is associated with epilepsy. Depression in epilepsy can change the response to treatment, aggravate the condition, reduce the quality of life, and increase the risk of suicidal tendencies among patients with epilepsy [61]. A study reported that major depression was associated with a sixfold increased risk of unprovoked seizures (95% CI, 1.56–22) [67].

This work focused more on the relationship between epilepsy and depression and the risk factors for depression in patients with epilepsy. Our study found that people pay more attention to the prevalence of depression in epilepsy than that epilepsy in depression. Moreover, it has been reported that epilepsy and depression share a common pathogenic mechanism [7]; thus, we believe that our study has implications for clinical work.

There are some limitations in this article. A wide variety of age ranges from 0.03 to 96 were sampled; this decreased the number of studies that could be pooled for further analyses. The studies had varied clinical diagnostic criteria used for depression or epilepsy. MINI is most frequently used in diagnosis of depression as gold standard [41]. Some studies suggested that PHQ-9, NDDI-E and HAMD did not differ statistically from MINI in the diagnosis of depression [23,41]. No studies have yet reported whether statistical differences exist between different diagnostic methods of epilepsy—this may influence the rate of depression in epilepsy or vice versa.

## Conclusion

Our study found that epilepsy was associated with an increased risk of depression. We worked with a limited number of studies, and their number was unevenly distributed among the three groups (depression in epilepsy, epilepsy in depression, and comorbidity); however, we can still draw some conclusions. Epilepsy is associated with an incidence of depression, and depression is associated with the severity of epilepsy. We thus need pay more attention to mental health for patients with epilepsy. The treatment of depression requires a more positive method, and interpretation of this meta-analysis requires caution. There was a large heterogeneity among the studies, and it may influence our results. More studies are needed in distinct populations and with accurate estimates to inform public health policy and prevention. This can help define health resource needs in these populations.

## Supporting information

S1 File Supplementary material: Search strategies, depression and epilepsy, and final search. (PDF)

S2 File PRISMA 2009 checklist. (PDF)
