# Supplementary material for: Exploring the association between epilepsy and depression: A systematic review and meta-analysis
Source: PLoS One. 2022 Dec 15;17(12):e0278907. doi: 10.1371/journal.pone.0278907 (PMC9754200; doi:10.1371/journal.pone.0278907)
Supplement: S1 File — (PDF) [file pone.0278907.s001.pdf]

S1 Appendix A. Search terms

Epilepsy	Depression
International database (e.g. PubMed)	
Epilepsies; Seizure Disorder; Seizure; Disorders; Awakening Epilepsy; Epilepsy, Awakening; Epileptic; Epilepticus; Epilepsy, Cryptogenic; Cryptogenic Epilepsies; Cryptogenic Epilepsy; Epilepsies, Cryptogenic; Aura; Auras	Depression; Depressive; Depressed; Depressive Symptoms; Depressive Symptom; Symptom, Depressive; Symptoms, Depressive; Emotional Depression; Depression, Emotional;
Chinese database (e.g. CNKI)	
“癫痫”、“痫证”、“羊角风”、“惊厥”、“难治性癫痫”、“癫痫持续状态”、“癫痫发作”、“颞叶癫痫”、“痫样放电”	“抑郁”、“郁证”、“抑郁症”、“抑郁症状”、“抑郁情绪”、“抑郁自评量表”

S1 Appendix B. Example search strategy: PubMed

1	((((((((((((Epilepsy[MeSH Terms]) OR (Epilepsies[Title/Abstract])) OR (Seizure Disorder[Title/Abstract])) OR (Seizure Disorders[Title/Abstract])) OR (Awakening Epilepsy[Title/Abstract])) OR (Epilepsy, Awakening[Title/Abstract])) OR (Epilepsy, Cryptogenic[Title/Abstract])) OR (Cryptogenic Epilepsies[Title/Abstract])) OR (Cryptogenic Epilepsy[Title/Abstract])) OR (Epilepsies, Cryptogenic[Title/Abstract])) OR (Aura[Title/Abstract])) OR (Auras[Title/Abstract])) OR (Epileptic[Title/Abstract])) OR (Epilepticus[Title/Abstract])
2	((((((((Depression[MeSH Terms]) OR (Depressive Symptoms[Title/Abstract])) OR (Depressive Symptom[Title/Abstract])) OR (Symptom, Depressive[Title/Abstract])) OR (Symptoms, Depressive[Title/Abstract])) OR (Emotional Depression[Title/Abstract])) OR (Depression, Emotional[Title/Abstract])) OR (Depressive[Title/Abstract])) OR (Depressed[Title/Abstract])
3	1 AND 2
